# LASSO-Nomogram model for ultrasound-atherosclerosis correlation and diagnostic verification in anterior circulation

**DOI:** 10.3389/fneur.2025.1639160

**Published:** 2025-10-13

**Authors:** Yi Liu, Pan Cheng, Xinying Jia, Delin Yu

**Affiliations:** ^1^Department of Ultrasound, Tianjin Huanhu Hospital, Tianjin, China; ^2^Department of Ultrasound, China-Japan Friendship Hospital, Beijing, China

**Keywords:** anterior circulation cerebral atherosclerosis, color Doppler ultrasonography of cervical vessels, transcranial Doppler ultrasonography, LASSO regression, nomogram model, correlation, diagnostic model

## Abstract

**Objective:**

This study aimed to investigate the relationship between cervical vascular ultrasound/transcranial Doppler ultrasound (TCD) parameters and anterior circulation cerebral atherosclerosis severity, and to develop a LASSO-Nomogram predictive model for clinical assessment.

**Methods:**

We retrospectively analyzed 350 patients with anterior circulation atherosclerosis, randomly divided into training (*n* = 245) and validation (*n* = 105) sets. Collected data included: (1) demographics and medical history; (2) lipid profiles; (3) ultrasound parameters [carotid intima-media thickness (IMT), plaque stability, internal carotid artery stenosis rate, middle cerebral artery peak systolic velocity (MCA-PSV), end-diastolic velocity (MCA-EDV), pulsatility index (PI), resistance index (RI)]. Patients were stratified by atherosclerosis severity (mild-moderate vs. severe). LASSO regression identified key predictors for nomogram prediction model construction, with model performance rigorously evaluated.

**Results:**

Baseline characteristics were balanced between training set and validation set (*p* > 0.05). Univariate analysis identified 10 significant factors (all *p* < 0.05). LASSO regression selected 9 key predictors: age, high-density lipoprotein (HDL), low-density lipoprotein (LDL), carotid IMT, plaque stability, stenosis rate, and MCA hemodynamics (PSV, EDV, RI). Multivariate analysis showed HDL (OR = 7.410) and stable plaques (OR = 3.987) as protective factors of arteriosclerosis, while LDL (OR = 0.621), carotid IMT (OR = 0.038), MCA-PSV (OR = 0.978), MCA-EDV (OR = 0.960), and RI (OR = 0.010) were risk factors of arteriosclerosis (all *p* < 0.05). The model demonstrated excellent discrimination (training C-index = 0.850; validation = 0.796) with AUCs of 0.849 (95% CI: 0.792–0.907) and 0.801 (95% CI: 0.698–0.904), respectively. Decision curve analysis confirmed clinical utility across threshold probabilities of 10–80%.

**Conclusion:**

Cervical vascular ultrasound and TCD parameters effectively reflect anterior circulation atherosclerosis severity. Our LASSO-Nomogram model provides clinicians with a reliable, visualized tool for individualized risk assessment, potentially improving patient management.

## Introduction

Anterior circulation cerebral atherosclerosis is the core cause of ischemic stroke, and its severity directly influences clinical decision-making and patient prognosis. Due to its high incidence, disability rate, and mortality, ischemic stroke has become a major public health issue. Early and accurate assessment of atherosclerosis is crucial for stroke prevention (1). Among traditional assessment methods, CT angiography (CTA) poses a radiation risk, and digital subtraction angiography (DSA), as an invasive examination, is accompanied by complications such as bleeding and vascular injury, and it is also costly, which limits its wide clinical application (2). In contrast, cervical vascular ultrasound and transcranial Doppler ultrasound (TCD) have become the mainstream screening tools due to their non-invasive and repeatable advantages. Cervical vascular ultrasound can clearly show the intima-media thickness of the carotid artery and plaque characteristics, and TCD can reflect the functional status of cerebral arteries through parameters such as blood flow velocity and resistance index. Both play important roles in early screening (3). In recent years, predictive models for head and neck atherosclerotic plaques have achieved notable progress. A study utilized routine health examinations and blood biomarkers to develop machine learning models for predicting the occurrence of carotid artery plaques. Among all six machine learning models, light GBM achieved the highest accuracy of 91.8%. Feature importance analysis revealed that age, low-density lipoprotein cholesterol (LDL-C), and systolic blood pressure were important predictive factors in the model (4). Another study developed an MRI-based radiomics model and found that it could accurately differentiate between symptomatic and asymptomatic carotid plaques, outperforming conventional models in identifying high-risk plaques (5). However, most existing studies rely on single imaging modalities or fail to fully integrate ultrasound and hemodynamic parameters, and there remains room for improvement in model visualization and clinical utility optimization. However, a single ultrasound index is difficult to comprehensively evaluate complex atherosclerotic lesions (6).

In this study, LASSO regression was innovatively combined with the Nomogram to construct a new evaluation model. LASSO regression can select key variables from multi-dimensional ultrasound indicators, avoid the problem of multicollinearity, and improve the stability of the model. The Nomogram can transform the complex model into a visual scoring tool, enabling clinicians to quickly quantify the atherosclerotic risk of patients and guide individualized treatment (7). Currently, there are few studies on the LASSO-Nomogram model based on ultrasound parameters in the field of cerebral artery assessment. In this study, by systematically analyzing the correlation between cervical vascular ultrasound and TCD indicators and anterior circulation lesions, this prediction model was constructed and verified. The aim was to break through the limitations of traditional assessment and provide a new path for dynamic monitoring of disease progression and optimization of intervention strategies. The research results are expected to promote the precise diagnosis and treatment of cerebrovascular diseases, reduce the social burden related to stroke, and have important clinical translational value.

## Materials and methods

### Study subjects

The clinical data of 350 patients with anterior circulation cerebral atherosclerosis who presented to the Department of Neurology in our hospital from January 2023 to December 2024 were retrospectively analyzed. The patients were randomly divided into a training set (*n* = 245) and a validation set (*n* = 105) at a ratio of 7:3. Inclusion criteria: patients were diagnosed with anterior circulation cerebral atherosclerosis (stenosis of the internal carotid artery, middle cerebral artery, or anterior cerebral artery ≥50%) by cranial CTA/MRA; all patients underwent cervical vascular color Doppler ultrasound and TCD examinations before surgery; the clinical data were complete, including demographic characteristics, medical history, blood lipid indices, and ultrasound parameters; the age was ≥40 years. Exclusion criteria: patients with severe dysfunction of the heart, liver, or kidney; patients with a history of stroke or transient ischemic attack (TIA); patients with cervical deformities or vascular diseases that could affect the accuracy of ultrasound examinations.

### Data collection

#### Demographic and medical history data

Age, gender, history of hypertension (systolic blood pressure ≥140 mmHg and/or diastolic blood pressure ≥90 mmHg, or currently taking antihypertensive drugs), history of diabetes mellitus (fasting blood glucose ≥7.0 mmol/L, or currently taking hypoglycemic drugs), history of hyperlipidemia (LDL-C ≥3.4 mmol/L, or currently taking lipid-regulating drugs), and smoking history (smoking an average of ≥1 cigarette per day for ≥1 year).

### Lipid indicators

Fasting serum levels of high-density lipoprotein (HDL), low-density lipoprotein (LDL) and total cholesterol (TC) were measured by the clinical laboratory of our hospital using an automatic biochemical analyzer.

### Color Doppler ultrasound parameters of cervical blood vessels

Carotid intima-media thickness (IMT) (the average value of IMT within 1 cm at the bilateral carotid bifurcations was taken), plaque stability (stable plaques: fibrous or calcified plaques; unstable plaques: lipid-deposited or ulcerated plaques), and internal carotid artery stenosis rate [calculated according to the criteria of the North American Symptomatic Carotid Endarterectomy Trial (NASCET)].

### Transcranial Doppler examination

The mean values of middle cerebral artery peak systolic velocity (MCA-PSV), middle cerebral artery end-diastolic velocity (MCA-EDV), pulsatility index (PI), and resistance index (RI) were obtained from the bilateral middle cerebral arteries.

### Staging of the degree of atherosclerosis

With reference to the Chinese Guidelines for the Diagnosis and Treatment of Cerebral Atherosclerosis (8), patients were divided into a mild-to-moderate group (stenosis rate of 50–70%, or stable plaques) and a severe group (stenosis rate >70%, or unstable plaques with a stenosis rate ≥50%) according to the vascular stenosis rate and plaque stability.

### Statistical analysis

Data pre-processing: Analysis was performed using SPSS 26.0 and R language 4.5.3. Continuous variables were expressed as “x ± s,” and independent-sample *t*-tests were used for comparisons between groups. Categorical variables were expressed as “number of cases (%),” and *χ*^2^ test were used for comparisons between groups. Variables screening: Key factors associated with the degree of atherosclerosis were selected using LASSO regression (R package “glmnet,” family = “binomial”). The optimal regularization parameter *λ* was determined through 10-fold cross-validation, selecting the *λ* value corresponding to the minimum cross-validation error (*λ*.min = 0.023). Multivariate logistic regression analysis to further identify the independent key factors, and their odds ratios (OR) and 95% confidence intervals (CI) were calculated. Variance inflation factors (VIF) were calculated to exclude multicollinearity (VIF threshold <10). Based on the finally determined independent factors, a nomogram model was constructed. To further validate the stability of the cross-validation results and minimize the impact of random variability on variable selection, this study employed the bootstrap resampling method for internal validation. By performing 1,000 repeated resamplings with replacement, bootstrap sample sets were generated to calculate the calibration slope and calibration intercept of the model, thereby assessing its performance in terms of overfitting and the agreement between predicted and actual probabilities. Model evaluation: Discrimination: The C-index was calculated, and the discriminatory ability of the model for the degree of sclerosis was evaluated in combination with the receiver operating characteristic (ROC) curve and area under curve (AUC). Calibration: A calibration curve was plotted to compare the consistency between the predicted probability and the actual probability, and the Hosmer–Lemeshow test was used to evaluate the goodness of fit. Clinical utility: The net benefit of the model at different threshold probabilities was evaluated through decision curve analysis (DCA). All code used for data processing and statistical analysis, along with an anonymized dataset sufficient to reproduce the main findings, have been provided as Supplementary material. Detailed descriptions of the supplementary files are available in the Supplementary material.

## Results

### Comparison of general clinical characteristics of patients in the training set and the validation set

A total of 350 patients with anterior circulation atherosclerosis, who randomly divided into training (*n* = 245) and validation (*n* = 105) sets, were included. No significant differences were found between the two sets in terms of age, gender, history of hypertension, history of diabetes, history of hyperlipidemia, smoking history, blood lipid indices, and ultrasound parameters (all *p* > 0.05), indicating a balanced grouping (Table 1).

**Table 1:** Comparison of general clinical characteristics of patients in the training set and the validation set.

Indicator	Classification	Training set (*n* = 245)	Validation set (*n* = 105)	*χ*^2^/*t*	*p*
Age (years)		62.81 ± 8.88	62.89 ± 7.68	0.081	0.936
Gender	Male	152 (62.04)	70 (66.67)	0.678	0.411
	Female	93 (37.96)	35 (33.33)		
History of hypertension	Yes	178 (72.65)	80 (76.19)	0.475	0.491
	No	67 (27.35)	25 (23.81)		
History of diabetes	Yes	91 (37.14)	40 (38.10)	0.029	0.866
	No	154 (62.86)	65 (61.90)		
History of hyperlipidemia	Yes	121 (49.39)	50 (47.62)	0.092	0.762
	No	124 (50.61)	55 (52.38)		
History of smoking	Yes	112 (45.71)	43 (40.95)	0.675	0.411
	No	133 (54.29)	62 (59.05)		
HDL (mmol/L)		1.26 ± 0.38	1.28 ± 0.41	0.441	0.659
LDL (mmol/L)		3.89 ± 0.96	3.85 ± 0.63	0.392	0.695
TC (mmol/L)		5.15 ± 1.18	5.12 ± 1.20	0.217	0.828
Carotid intima-media thickness (mm)		1.18 ± 0.20	1.20 ± 0.22	0.832	0.406
Plaque stability	Stable	142 (57.96)	65 (61.90)	0.474	0.491
	Unstable	103 (42.04)	40 (38.10)		
Internal carotid artery stenosis rate (%)		57.51 ± 9.98	56.58 ± 10.01	0.798	0.425
MCA-PSV (cm/s)		110.31 ± 25.41	113.52 ± 26.55	1.068	0.286
MCA-EDV (cm/s)		60.51 ± 11.28	60.55 ± 10.98	0.031	0.976
PI		1.02 ± 0.19	1.05 ± 0.23	1.268	0.205
RI		0.58 ± 0.09	0.60 ± 0.11	1.778	0.076

### Univariate analysis of factors related to the degree of atherosclerotic lesions in the anterior circulation cerebral arteries in the training set

In the training set, significant differences (*p* < 0.05) were found between the mild-moderate atherosclerosis group and the severe atherosclerosis group in terms of age, HDL, LDL, TC, carotid IMT, plaque stability, internal carotid artery stenosis rate, MCA-PSV, MCA-EDV, PI and RI. No collinearity issues were detected among the variables through tests (tolerance >0.1, variance inflation factor (VIF) <10, condition index <30, and no situation where the variance proportions of multiple covariates under the same eigenvalue were >50%) (Table 2).

**Table 2:** Univariate analysis of factors related to the degree of atherosclerotic lesions in the anterior circulation cerebral arteries in the training set.

Indicator	Classification	The mild–moderate sclerosis group (*n* = 143)	The severe sclerosis group (*n* = 102)	*χ*^2^/*t*	*p*
Age (years)			63.58 ± 8.89	2.445	0.015
Gender	Male	85 (59.44)	67 (65.68)	0.986	0.321
	Female	58 (40.56)	35 (34.32)		
History of hypertension	Yes	98 (68.53)	80 (78.43)	2.937	0.086
	No	45 (31.47)	22 (21.57)		
History of diabetes	Yes	50 (34.96)	41 (40.19)	0.698	0.404
	No	93 (65.04)	61 (59.81)		
History of hyperlipidemia	Yes	65 (45.45)	56 (54.90)	2.126	0.145
	No	78 (54.55)	46 (45.10)		
History of smoking	Yes	58 (40.56)	54 (52.94)	3.678	0.055
	No	85 (59.44)	48 (47.06)		
HDL (mmol/L)		1.32 ± 0.30	1.16 ± 0.33	3.946	0.001
LDL (mmol/L)		3.62 ± 0.98	4.01 ± 0.95	3.109	0.002
TC (mmol/L)		4.95 ± 1.15	5.30 ± 1.28	2.304	0.022
Carotid intima-media thickness (mm)		1.01 ± 0.25	1.25 ± 0.31	2.511	0.013
Plaque stability	Stable	98 (68.53)	44 (43.14)	15.756	0.001
	Unstable	45 (31.47)	58 (56.86)		
Internal carotid artery stenosis rate (%)		55.51 ± 9.72	58.81 ± 12.32	2.341	0.02
MCA-PSV (cm/s)		105.31 ± 28.41	115.23 ± 25.72	2.801	0.005
MCA-EDV (cm/s)		58.51 ± 10.21	63.21 ± 13.52	3.099	0.002
PI		0.99 ± 0.18	1.05 ± 0.22	2.343	0.020
RI		0.57 ± 0.08	0.6 ± 0.10	2.605	0.010

### Results of LASSO regression analysis

**Figure 1:**
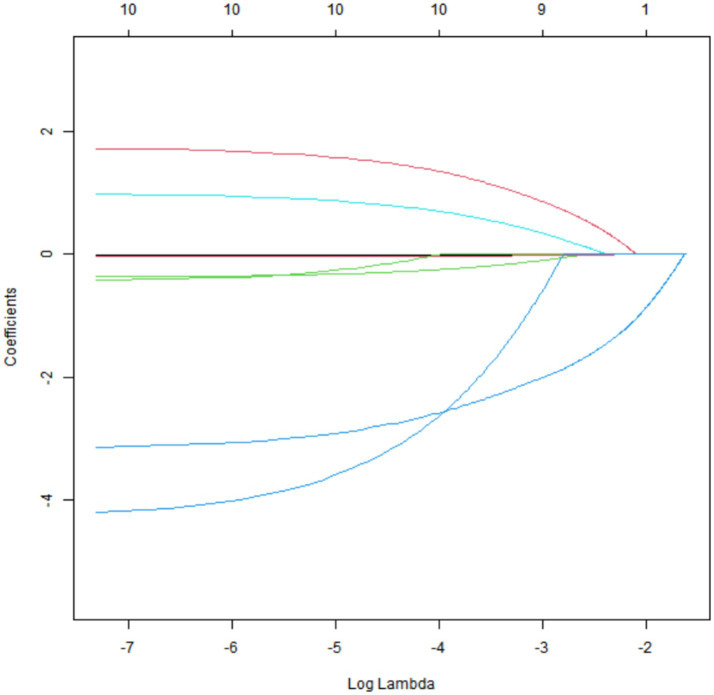
Coefficient path of LASSO regression.

**Figure 2:**
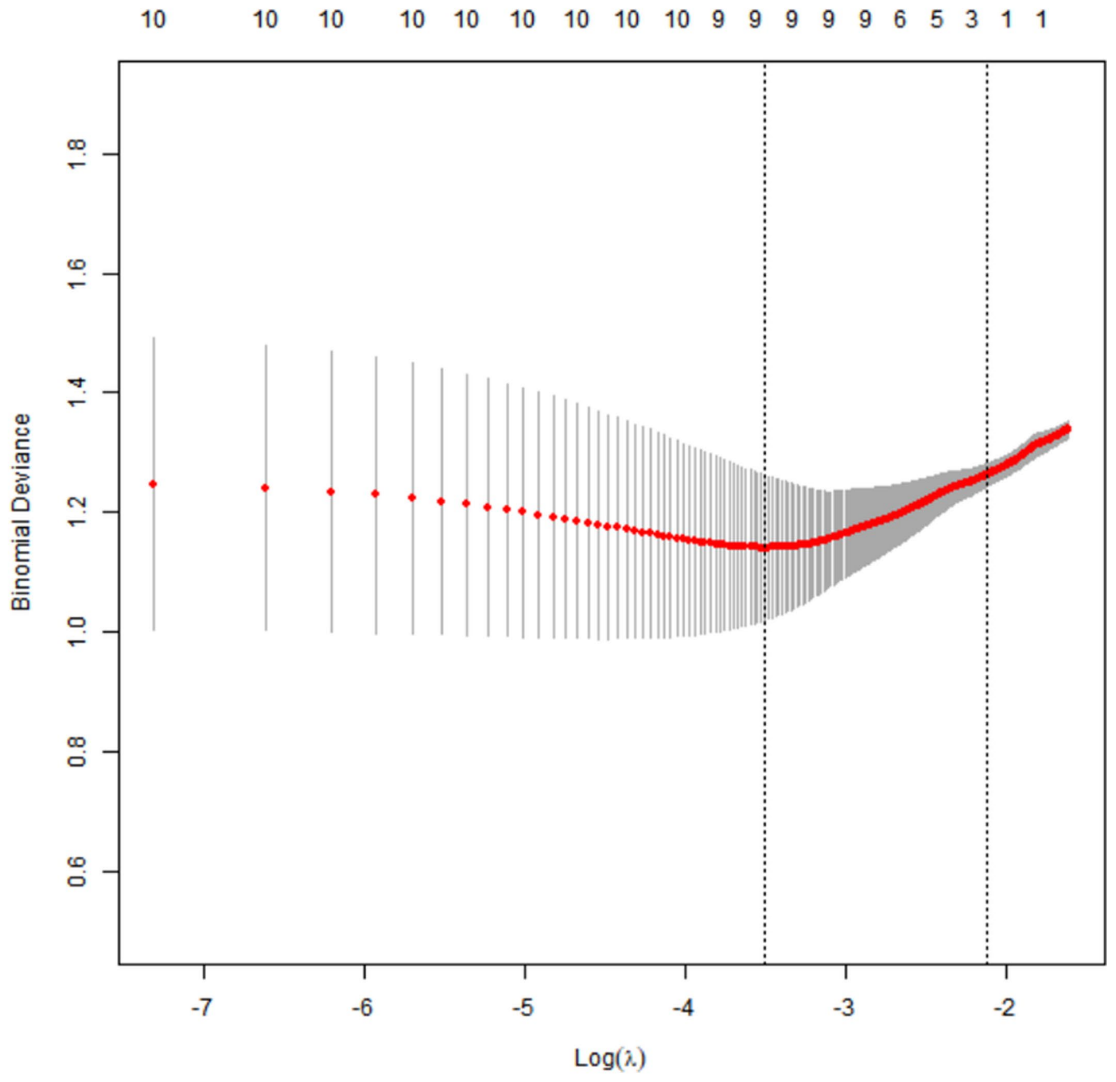
Results of LASSO regression cross-validation.

Through 10-fold cross-validation, 9 key factors were selected from 11 candidate variables by LASSO regression: age, HDL, LDL, carotid IMT, plaque stability, internal carotid artery stenosis rate, MCA-PSV, MCA-EDV, and RI (Figures 1, 2).

### Multivariate logistic regression analysis of the degree of atherosclerotic lesions in the anterior cerebral circulation arteries

Multivariate logistic regression was performed with variables screened by LASSO regression as independent variables and the degree of atherosclerotic lesions as the dependent variable (Table 3). The results showed that high level of HDL and stable plaque stability were associated with increased odds for mild-moderate arteriosclerosis (all *p* < 0.05), indicating high level of HDL and stable plaque stability as protective factors of arteriosclerosis. On the contrary, high level of LDL, carotid IMT, MCA-PSV, MCA-EDV, and RI associated with decreased odds for mild-moderate arteriosclerosis (all *p* < 0.05), indicating above indicators as risk factors of arteriosclerosis (Table 4).

**Table 3:** Variable assignment methods.

Variables	Meaning	Assignment
X1	HDL	Continuous variable
X2	LDL	Continuous variable
X3	Carotid intima-media thickness	Continuous variable
X4	Plaque stability	1 = stable, 0 = unstable
X5	MCA-PSV	Continuous variable
X6	MCA-EDV	Continuous variable
X7	RI	Continuous variable
Y	Degree of arteriosclerosis	1 = the mild-moderate arteriosclerosis, 0 = the severe arteriosclerosis

**Table 4:** Logistic regression analysis of the degree of atherosclerosis in the anterior cerebral circulation arteries.

Item	*B*	Standard error	Wald	*p*	OR	95% CI
HDL	2.003	0.553	13.131	0.001	7.410	2.508–21.893
LDL	−0.476	0.178	7.175	0.007	0.621	0.438–0.880
Carotid intima-media thickness	−3.265	0.655	24.861	0.001	0.038	0.011–0.138
Plaque stability	1.383	0.339	16.627	0.001	3.987	2.051–7.751
MCA-PSV	−0.022	0.006	12.731	0.001	0.978	0.966–0.990
MCA-EDV	−0.041	0.015	7.790	0.005	0.960	0.932–0.988
RI	−4.613	1.863	6.131	0.013	0.010	0.001–0.392

### The correlation between color ultrasonography of cervical vessels and transcranial Doppler ultrasonography and the degree of anterior circulation cerebral atherosclerosis

A significant negative correlation was found between IMT and the degree of anterior circulation cerebral atherosclerosis (Pearson correlation coefficient was −0.399, *p* < 0.01). That is, the thicker the carotid IMT, the more severe the degree of atherosclerosis. Meanwhile, it also showed a certain correlation with a history of hyperlipidemia and a history of smoking (*p* < 0.05). The nature of plaques was significantly correlated with the degree of atherosclerosis, gender, a history of hypertension, a history of diabetes, a history of hyperlipidemia, and a history of smoking (*p* < 0.01). The stenosis rate of the internal carotid artery was weakly correlated with age and the degree of atherosclerosis (*p* < 0.05). There were significant correlations between MCA-PSV, MCA-EDV, PI, and RI of the middle cerebral artery and the degree of anterior circulation cerebral atherosclerosis (*p* < 0.05) (Table 5).

**Table 5:** The correlation between color ultrasonography of cervical vessels and transcranial Doppler ultrasonography and the degree of anterior circulation cerebral atherosclerosis.

Factors		Age	Gender	History of hypertension	History of diabetes	History of hyperlipidemia	History of smoking	Degree of arteriosclerosis
Carotid intima-media	Pearson’s correlation	−0.002	0.051	0.004	0.095	0.151^*^	0.135^*^	−0.399^**^
	Sig.	0.978	0.426	0.949	0.137	0.018	0.035	0.001
Plaque stability	Pearson’s correlation	−0.104	0.697^**^	0.720^**^	0.655^**^	0.643^**^	0.616^**^	0.254^**^
	Sig.	0.106	0.001	0.001	0.001	0.001	0.001	0.001
Internal carotid artery stenosis rate	Pearson’s correlation	0.147^*^	0.030	−0.002	−0.034	−0.021	−0.044	−0.148^*^
	Sig.	0.021	0.635	0.973	0.592	0.744	0.494	0.020
MCA-PSV	Pearson’s correlation	0.033	0.076	0.090	0.106	0.094	0.123	−0.177^**^
	Sig.	0.611	0.237	0.160	0.098	0.142	0.054	0.006
MCA-EDV	Pearson’s correlation	0.070	0.059	0.114	−0.056	−0.021	−0.037	−0.195^**^
	Sig.	0.273	0.358	0.075	0.386	0.747	0.569	0.002
RI	Pearson’s correlation	0.037	0.051	0.066	0.001	0.020	0.002	−0.140^*^
	Sig.	0.560	0.431	0.306	0.991	0.751	0.970	0.028

^*^p < 0.05 and ^**^p < 0.01.

### Nomogram prediction model construction and evaluation

**Figure 3:**
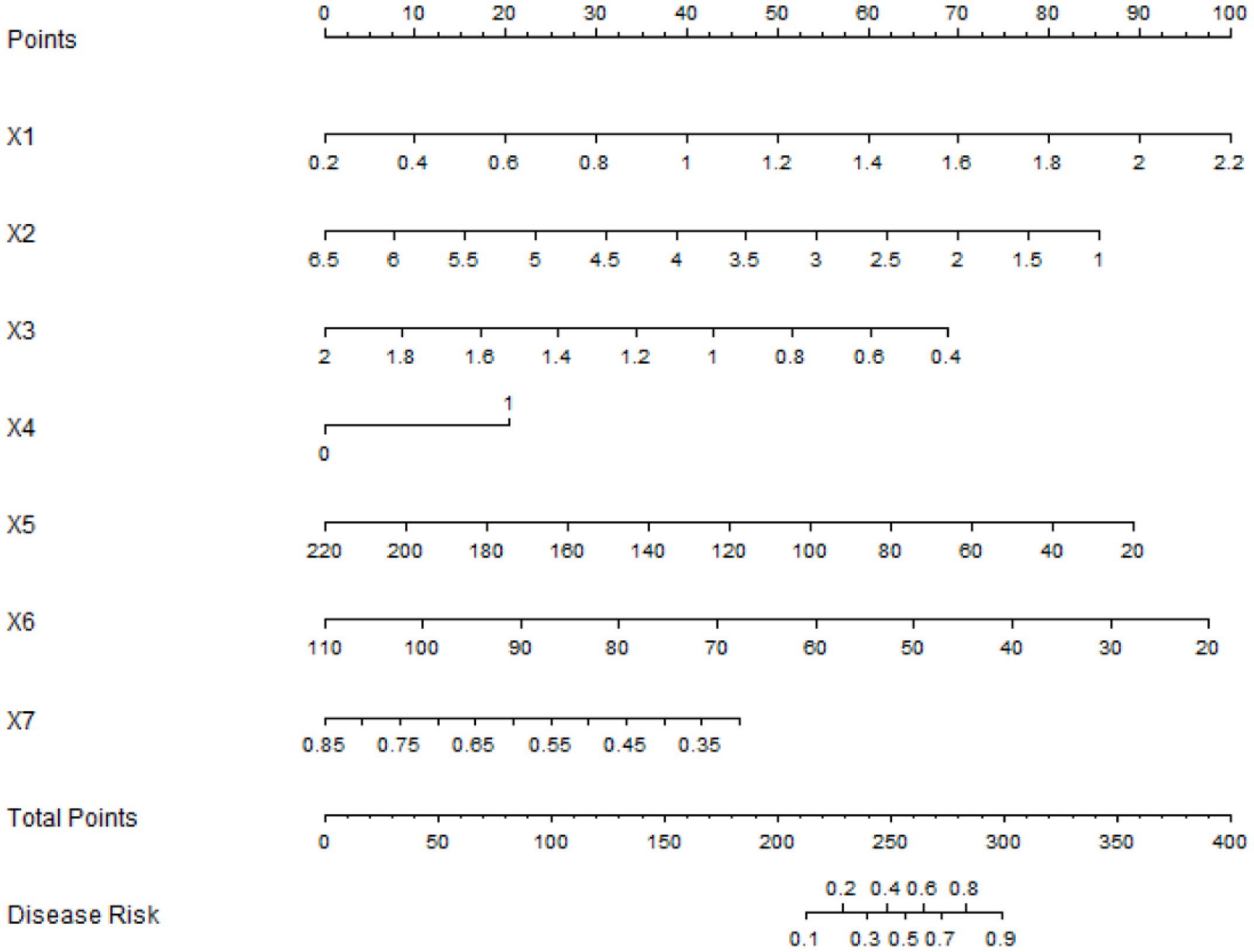
Nomogram prediction model. X1: HDL; X2: LDL; X3: carotid intima-media; X4: plaque stability; X5: MCA-PSV; X6: MCA-EDV; X7: RI.

**Figure 4:**
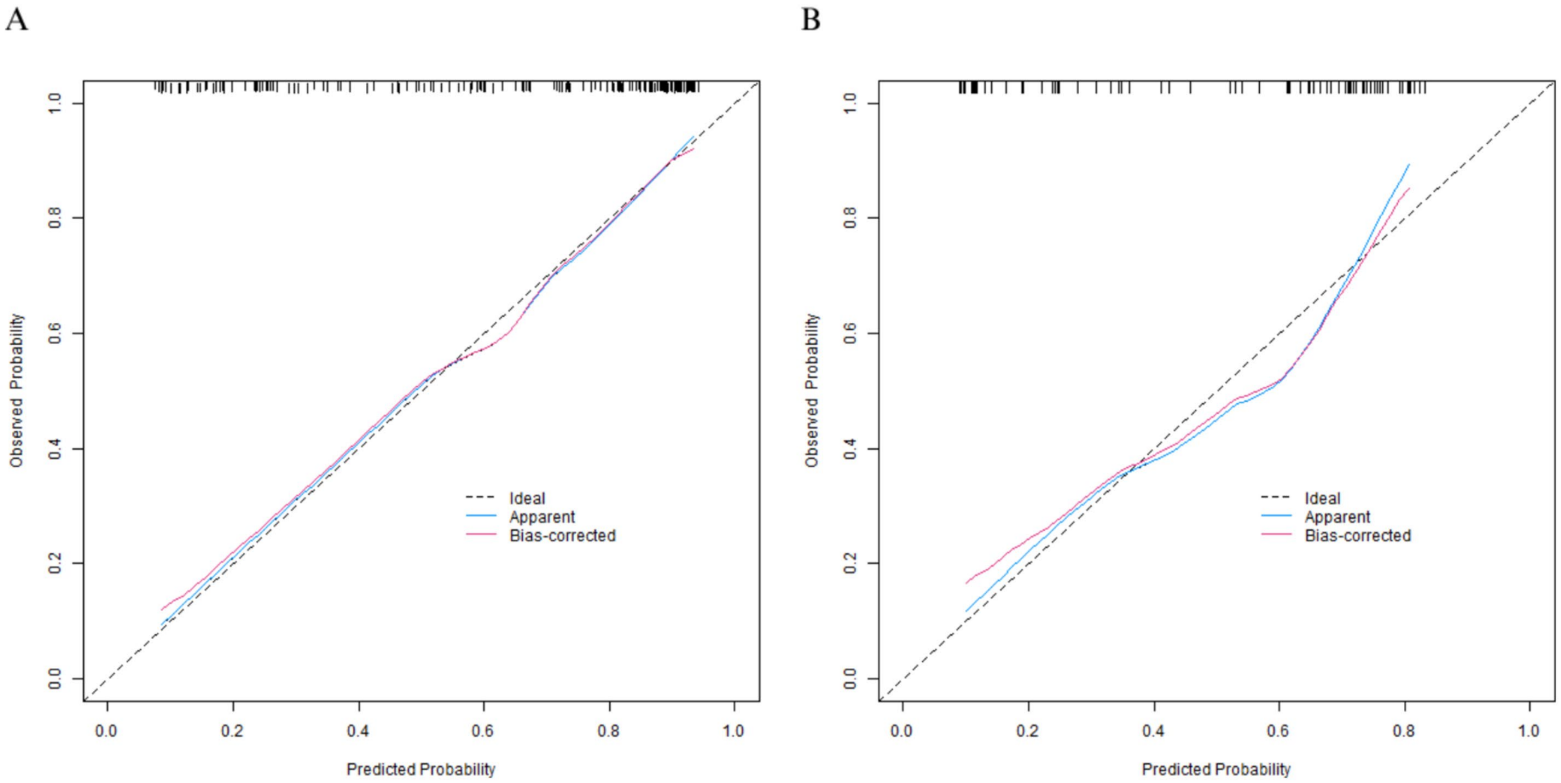
Calibration curves (**A**: the training set, **B**: the validation set).

**Figure 5:**
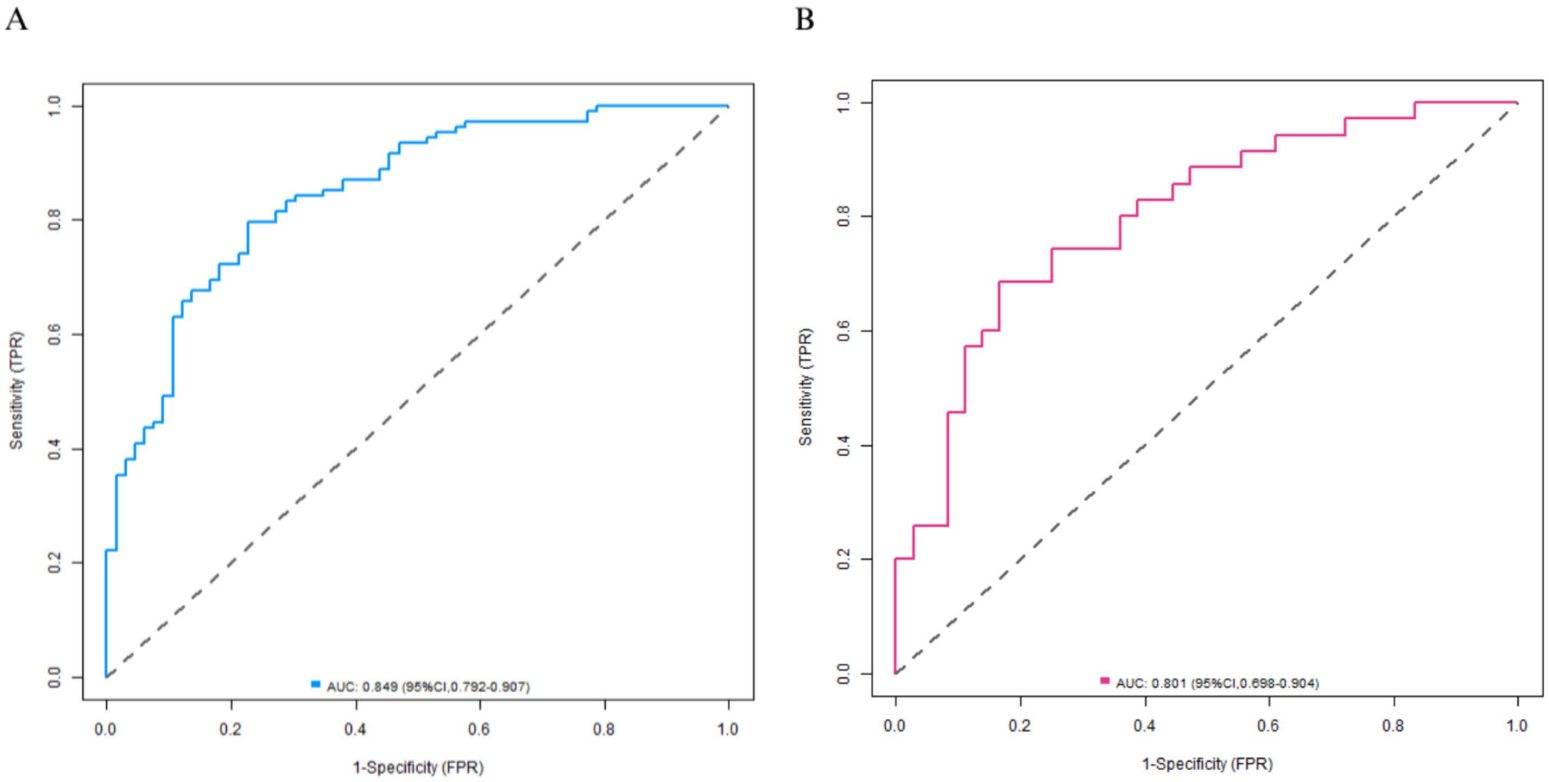
Receiver operating characteristic curves (**A**: the training set, **B**: the validation set).

**Figure 6:**
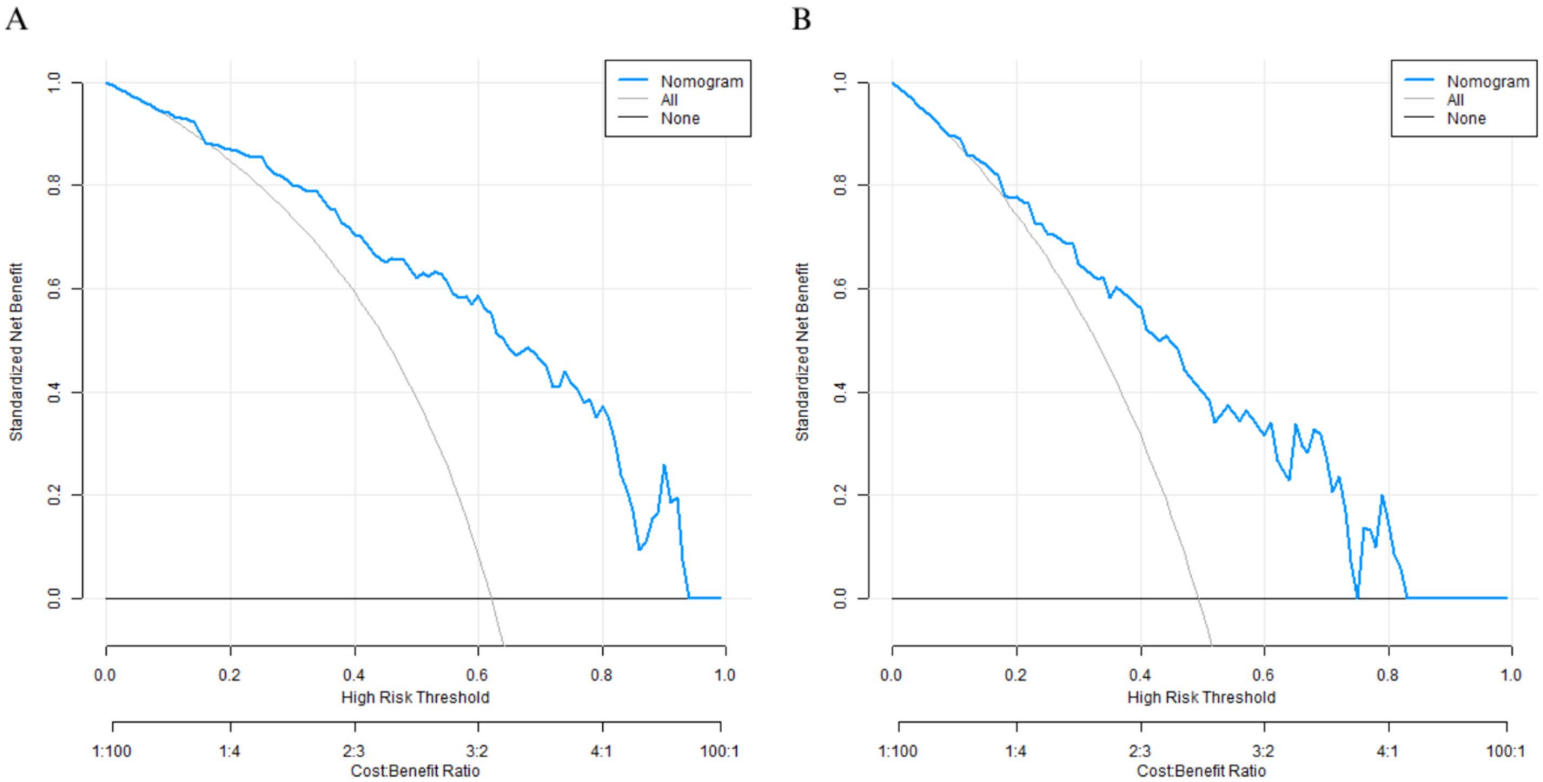
Decision curves (**A**: the training set, **B**: the validation set).

A Nomogram was constructed based on the logistic regression coefficients (Figure 3). Each variable corresponded to a score, and a higher total score indicated a higher probability of mild-moderate sclerosis. The C-indices of the training set and the validation set were 0.850 and 0.796, respectively. The calibration curve showed a high consistency between the predicted values and the actual values (Figure 4). The Hosmer–Lemeshow test yielded a *p* > 0.05, indicating a good fit of the model. The ROC curve showed that the AUC of the training set was 0.849 and that of the validation set was 0.801 (Figure 5). The decision curve indicated that the model had a better net benefit than the “all severe” or “all mild-moderate” strategies when the threshold probability ranged from 0.10 to 0.80 (Figure 6). The bootstrap calibration results showed that the calibration slope was 0.951 (95% CI: 0.912–0.989) with an intercept of −0.032 (95% CI: −0.071 to 0.007) in the training set, while in the validation set, the slope was 0.893 (95% CI: 0.832–0.954) with an intercept of 0.045 (95% CI: −0.011 to 0.101). Slopes close to 1 and intercepts near 0 indicate that the model has good calibration ability, with highly consistent predicted and actual risks, and no significant overfitting was observed.

### Incremental value analysis of the model

To evaluate the clinical advantages of the nomogram model, this study compared its performance with that of a single ultrasound marker model, a traditional risk factor model, and a combined “ultrasound + traditional” model. The results showed that C-indices of nomogram predictive model in the training and validation sets were highest (Table 6).

**Table 6:** Performance comparison of different predictive models in the training and validation sets.

Model type	Training set AUC (95% CI)	Validation set AUC (95% CI)	Training set C-index	Validation set C-index
Traditional risk factor model (age + HDL + LDL)	0.716 (0.638–0.793)	0.653 (0.515–0.792)	0.717	0.659
Single ultrasound marker model (carotid IMT)	0.742 (0.665–0.819)	0.750 (0.622–0.878)	0.747	0.756
Single ultrasound marker model (MCA-PSV)	0.572 (0.486–0.658)	0.665 (0.534–0.796)	0.571	0.666
Combined “traditional + ultrasound” model (age + HDL + LDL + carotid IMT + MCA-PSV)	0.660 (0.577–0.743)	0.744 (0.619–0.868)	0.656	0.757
Nomogram model	0.849 (0.792–0.907)	0.801 (0.698–0.904)	0.850	0.796

## Discussion

As the core pathological basis of ischemic stroke, early and accurate assessment of anterior circulation cerebral atherosclerosis is crucial for stroke prevention and treatment strategy optimization (9). Although traditional imaging methods such as CTA and DSA can visually display vascular lesions, the radiation risk of CTA and the invasiveness and high cost of DSA limit their widespread application in screening and dynamic monitoring (10). In contrast, TCD has become the preferred tool for the initial clinical assessment of cerebrovascular lesions due to its advantages of being non-invasive, repeatable, and cost-effective. Carotid vascular ultrasound can quantify the IMT, identify the nature of plaques, and evaluate the stenosis rate of the internal carotid artery, while TCD reflects the hemodynamic status of cerebral arteries through the blood flow parameters of the middle cerebral artery (such as the peak systolic velocity of the middle cerebral artery, MCA-PSV, and the resistance index, RI) (11). However, atherosclerosis is a complex pathological process involving multiple factors. A single ultrasound index is difficult to comprehensively capture the degree of lesions, and in clinical practice, problems such as strong subjectivity in index screening and insufficient generalization ability of the model are often encountered. This study focuses on integrating multi-dimensional indicators of carotid vascular ultrasound and TCD. By combining LASSO regression with the Nomogram model, an objective data-driven accurate assessment tool is aimed to be constructed. LASSO regression can screen key factors from numerous candidate variables through regularization methods, effectively solve the problem of multicollinearity, and improve the stability of the model; Nomogram transforms the complex statistical model into a visual nomogram, facilitating clinicians to quickly quantify the patient’s risk. Currently, there is a lack of high-quality research on the comprehensive assessment model of ultrasound indicators for anterior circulation cerebral atherosclerosis. In this study, through a systematic analysis of the clinical data of 350 patients, the correlation between multimodal ultrasound indicators and the degree of atherosclerosis was verified, and a diagnostic model with good predictive performance was constructed, providing a new approach to break through the limitations of traditional assessment (12).

The LASSO-Nomogram model constructed in this study demonstrates a complete logical chain from data screening to visual application. First, 9 key factors were screened out from 11 candidate variables through LASSO regression, including age, HDL, LDL, carotid artery IMT, plaque nature, internal carotid artery stenosis rate, MCA-PSV, MCA-EDV, and RI. In this process, the optimal regularization parameter was determined by 3-fold cross-validation to ensure the generalization ability of the model between the training set and the validation set and avoid overfitting. Subsequently, logistic regression analysis further clarified the independent predictive value of each index. Among them, HDL and stable plaques were protective factors, while LDL, carotid artery IMT, MCA-PSV, MCA-EDV, and RI were risk factors, providing a statistical basis for the weight assignment of the Nomogram.

In terms of model efficacy, the C-indices of the training set and the validation set were 0.850 and 0.796 respectively, and the AUC exceeded 0.8 for both, indicating that the model had a strong ability to distinguish between mild-moderate and severe arteriosclerosis. The calibration curve showed a high consistency between the predicted probability and the actual probability, and the *p*-value of the Hosmer–Lemeshow test was >0.05, suggesting that the model was well-fitted without significant bias. Decision curve analysis confirmed that within a wide range of threshold probabilities from 0.10 to 0.80, the net benefit of this model was better than the extreme strategies of “all severe” or “all mild-moderate,” indicating its high practical value in clinical practice.

To validate the clinical applicability of the model, a typical case was selected for demonstration: a 65-year-old male patient was admitted with “transient weakness of the right limb.” Carotid ultrasound revealed an IMT of 1.25 mm with unstable plaque formation, while TCD measurements showed an MCA-PSV of 125 cm/s, MCA-EDV of 110 cm/s and an RI of 0.62. Lipid profiling indicated an LDL level of 4.2 mmol/L and an HDL level of 1.1 mmol/L. These parameters were input into the Nomogram model, which calculated a total score corresponding to a 65% probability of mild-to-moderate atherosclerosis, indicating a high predicted risk of severe atherosclerosis. This prediction was consistent with subsequent DSA results (showing 75% stenosis of the left middle cerebral artery). This case demonstrates the model’s ability to integrate multimodal ultrasound and lipid indicators, providing clinicians with rapid and intuitive decision-making support.

Compared with traditional single-index evaluation, the advantage of the LASSO-Nomogram model lies in the integration and visual presentation of multi-dimensional data (13). For example, carotid artery IMT reflects the structural changes of the vascular wall, plaque nature reflects the stability of the lesion, and TCD parameters reveal hemodynamic abnormalities. The combination of the three can characterize the features of atherosclerosis from the three levels of structure, nature, and function (14). In addition, the blood lipid indices (HDL, LDL) included in the model not only reflect the metabolic state but also are directly related to the pathological mechanism of plaque formation, reflecting the synergistic effect of biological and imaging indices. The integration of such multi-modal data not only improves the prediction efficacy but also provides targets for individualized intervention, such as intensive lipid-lowering treatment for patients with high LDL and enhanced anti-platelet intervention for patients with unstable plaques.

It is worth noting that in this study, collinearity interference was excluded through strict variable screening (tolerance >0.1, VIF <10), ensuring the robustness of the model. Compared with the stepwise regression method commonly used in previous studies, LASSO regression has higher variable selection efficiency in high-dimensional data, especially suitable for complex data sets mainly composed of continuous variables such as ultrasound indices. The visual characteristics of the Nomogram further lower the threshold of clinical application. Doctors can quickly obtain the probability prediction of the degree of arteriosclerosis by accumulating the scores corresponding to each index. This “one-stop” evaluation tool has significant convenience advantages in outpatient screening and follow-up management.

The 7 core indices (HDL, LDL, carotid artery IMT, plaque nature, MCA-PSV, MCA-EDV, RI) screened in this study reveal the pathological mechanism of atherosclerosis from different dimensions, and their correlations can be interpreted in depth from the following aspects: as a “vascular protective factor,” the increase of HDL is negatively correlated with mild-moderate arteriosclerosis (OR = 7.410), which is in line with the classic pathological theory of atherosclerosis. HDL reduces lipid deposition through reverse cholesterol transport, inhibits the inflammatory response, and maintains vascular endothelial function (15). In this study, for every 1 mmol/L increase in HDL, the probability of mild-moderate arteriosclerosis increased by 7.41 times, suggesting that a good HDL level is an important protective factor for milder lesions (16).

On the contrary, as an “atherogenic factor,” the increase of LDL (OR = 0.621) reflects an increased risk of lipid infiltration into the vascular wall. LDL-C promotes the formation of foam cells through oxidative modification and is the core driving factor for plaque progression. Intensive management of LDL in clinical practice (such as statin intervention) can be regarded as a key link to block the deterioration of the lesion. Carotid artery IMT is a sensitive index reflecting early vascular wall lesions. In this study, thickening of IMT (OR = 0.038) was significantly correlated with severe arteriosclerosis, indicating that the degree of vascular wall remodeling is consistent with cerebral artery lesions (17). For every 1 mm increase in IMT, the probability of mild-moderate arteriosclerosis decreased to 3.8%, suggesting that IMT can be used as a window to evaluate the overall atherosclerotic burden (18).

The difference in plaque nature is directly related to the stability of the lesion: stable plaques (fibrous or calcified plaques) are protective factors (OR = 3.987). They have a complete fibrous cap and a small lipid core and are not prone to rupture and cause thrombosis. Unstable plaques (lipid-deposited or ulcerated plaques) are at high risk of ischemic events due to their high vulnerability and are often accompanied by the progression of the stenosis rate (19). The identification of plaque nature by ultrasound makes up for the deficiency of simple stenosis rate evaluation and provides a pathological basis for clinical hierarchical management (20).

MCA-PSV and MCA-EDV reflect the blood flow velocity in the middle cerebral artery. Their increase is negatively correlated with mild-moderate arteriosclerosis (OR values are 0.978 and 0.960 respectively), which may be related to the compensatory dilation of blood vessels. When there is mild stenosis, the blood flow velocity increases compensatorily to maintain cerebral perfusion, while when there is severe stenosis, the vascular compliance decreases and the flow velocity is inhibited (21). As a resistance index, the decrease of RI (OR = 0.010) indicates an increase in vascular resistance, which is related to the increase in arterial stiffness and microcirculation disorders and is an important sign of severe arteriosclerosis (22). The combination of the three can dynamically evaluate the functional state of cerebral arteries: an increase in RI reflects the decline of vascular elasticity, and abnormal MCA-PSV/EDV indicates hemodynamic decompensation, jointly constituting a progressive evaluation system from structural lesions to functional abnormalities (23).

These indices are not independent but are interrelated through pathological mechanisms: an increase in LDL leads to lipid deposition, causing thickening of IMT and plaque formation, which in turn affects the vascular diameter and hemodynamics (such as an increase in the stenosis rate leading to an increase in RI); HDL delays plaque progression and maintains vascular elasticity through anti-inflammatory and antioxidant effects (24). In clinical practice, the combined evaluation of these indices can achieve a three-dimensional diagnosis of “structure-nature-function.” For example, patients with increased LDL, unstable plaques, and abnormal RI should be vigilant against the risk of severe arteriosclerosis, and enhanced imaging follow-up is recommended. For patients with normal HDL, stable plaques, and stable blood flow parameters, lifestyle intervention can be prioritized (25). This multi-dimensional evaluation mode helps to break through the limitations of single indices and provides a basis for precise treatment.

Although this study constructed a LASSO-Nomogram model with good efficacy, there are still some limitations. Firstly, the study was a single-center retrospective design. Although the training set and the validation set were divided, external multi-center validation was not carried out, which may affect the universality of the model. The main reasons for not conducting external validation are as follows: (1) Limited by the data sharing mechanism during the study period, it was difficult to quickly obtain similar data from other centers; (2) The diagnosis of anterior circulation cerebral atherosclerosis depends on the gold standard of CTA/MRA. Furthermore, the absence of a healthy control group prevents us from establishing baseline values for our predictive parameters and from making direct comparisons between diseased and non-diseased states. Our model is therefore intended for use specifically in clinical populations already diagnosed with significant stenosis, rather than for primary screening. Secondly, our predictive model was limited to lipid profiles and ultrasound-derived parameters. We did not incorporate other potentially crucial prognostic factors, such as systemic inflammatory biomarkers (e.g., high-sensitivity C-reactive protein), detailed history of medication use (in particular, the dosage and adherence to statin therapy), or prior history of stroke or transient ischemic attack. The absence of these variables is a significant source of potential residual confounding. For instance, unmeasured differences in anti-inflammatory status or the intensity of lipid-lowering therapy could partly account for the observed associations, potentially leading to an over- or underestimation of the true effect of the variables we included. Therefore, while our model identifies informative markers, the findings should be interpreted in the context of these unmeasured confounders. Future studies aimed at developing a more comprehensive clinical prediction tool must prioritize the inclusion of these critical clinical data points. In addition, although this study employed LASSO regression for variable selection and incorporated seven predictors in the multivariate analysis with a total sample size of 350, the number of events per variable (EPV) may fall below the methodologically recommended threshold (EPV ≥10) under the condition of a roughly balanced distribution of the binary outcome. This limitation may somewhat affect the stability of model parameter estimation and its generalizability. Finally, our inclusion criteria were restricted to patients with moderate-to-severe anterior circulation stenosis (≥50%). While this focused approach allowed us to develop a model highly relevant to a high-risk clinical population, it consequently excludes individuals with early or mild atherosclerosis. Therefore, our model is not validated for and should not be used as a screening tool in the general or asymptomatic population. Its primary utility lies in risk stratification for patients who have already been identified with significant disease. Future research should aim to include a broader spectrum of patients, from those with no or mild stenosis to those with severe disease, to develop a more comprehensive model that can truly serve a screening purpose and understand the complete natural history of atherosclerotic progression.

Future research can be expanded in the following directions: (1) Conduct multi-center, prospective cohort studies, including patients from different regions and ethnic groups, to verify the external validity of the model; (2) Include healthy people and patients with non-atherosclerotic cerebrovascular diseases to expand the scope of application of the model; (3) Combine molecular biological indices (such as vascular endothelial growth factor and high-sensitivity C-reactive protein) with AI image analysis technology to further improve the prediction accuracy of the model; (4) Develop a clinical decision-support system based on the Nomogram to achieve real-time risk assessment through mobile tools and promote the implementation of precision medicine in primary medical institutions.

In conclusion, this study confirmed that the multi-dimensional indices of neck vascular ultrasound and TCD are closely related to the degree of anterior circulation cerebral atherosclerosis. The constructed LASSO-Nomogram model provides an efficient tool for clinical evaluation through data-driven variable screening and visual presentation. This model not only integrates the ultrasound features from the three levels of structure, nature, and function but also includes key biological indices, combining scientificity and practicality. Despite the single-center limitation, its innovative modeling idea and good prediction efficacy lay a foundation for the precise evaluation of cerebrovascular diseases. With the advancement of external validation and the integration of multi-modal data, the LASSO-Nomogram model is expected to become a core tool for dynamically monitoring the progression of atherosclerosis and guiding individualized intervention, helping to reduce the incidence and disability rate of ischemic stroke.

## Data Availability

The original contributions presented in the study are included in the article/Supplementary material, further inquiries can be directed to the corresponding author.
